# Multimodal Neuroimaging Predictors of Gait Decline

**DOI:** 10.1093/geroni/igaa057.2769

**Published:** 2020-12-16

**Authors:** Lana Sargent, Qu Tian, Mike Nalls, Andrew Singleton, Stephanie Studenski, Susan Resnick, Luigi Ferrucci

**Affiliations:** 1 Virginia Commonwealth University School of Nursing, Richmond, Virginia, United States; 2 National Institute on Aging, Bethesda, Maryland, United States; 3 University of Pittsburgh, Pittsburgh, Pennsylvania, United States

## Abstract

Prior studies suggesting associations between cortical brain areas and gait speed has been largely cross-sectional and limited to one modality neuroimaging. Using machine learning from 506 cognitively normal BLSA participants aged 55+ who had repeated measures of brain volumes, diffusion tensor imaging (DTI), and gait speed, we examined multimodal neuroimaging predictors of gait decline, accounting for demographics, body composition, and grip strength. Significant predictors of gait decline included changes in volumes and DTI measures of gray matter in selected frontal, parietal, temporal, and subcortical areas, as well as white matter changes in both fractional anisotropy and diffusivity of tracts connecting frontal areas to subcortical motor areas. This predictive model highlights the importance of atrophy and microstructural deterioration in selected frontal and subcortical motor areas in predicting gait speed decline.

